# Acute Psychosis Following Corticosteroid Administration

**DOI:** 10.7759/cureus.18093

**Published:** 2021-09-19

**Authors:** Sana Elham Kazi, Sheikh Hoque

**Affiliations:** 1 Psychiatry, Brookdale University Hospital Medical Center, Brooklyn, USA

**Keywords:** acute hypoxemic respiratory failure, covid, covid vaccine, corticosteroid, glucocorticoid, steroid induced psychosis, acute psychosis

## Abstract

Glucocorticoids are commonly used to treat endocrine as well as non-endocrine disorders. Unfortunately, these agents are associated with multiple adverse effects affecting various organ systems. A 55-year-old woman with type 2 diabetes mellitus and hypertension with no past psychiatric history was admitted to the hospital for acute hypoxic respiratory failure secondary to coronavirus disease 2019 (COVID-19) pneumonia. The patient did not exhibit any psychiatric symptoms during the initial admission. However, she was re-admitted three days after the initial discharge, presenting with acute psychosis following the intravenous dexamethasone administration for seven days. Neuropsychiatric effects of glucocorticoids include depression, mania, agitation, mood lability, anxiety, insomnia, catatonia, depersonalization, delirium, dementia, and psychosis. Clinicians should be aware of the acute neuropsychiatric side effects of corticosteroids and evaluate patients for delirium if clinically indicated. Further research is needed to identify the pathophysiology and predisposing factors contributing to neuropsychiatric side effects of corticosteroid administration. The use of atypical antipsychotics in the management of these sequelae needs to be explored as well.

## Introduction

Corticosteroids are endogenous steroid hormones produced in the adrenal cortex. There are two main classes of corticosteroids: mineralocorticoids and glucocorticoids. Mineralocorticoids are regulated by the renin-angiotensin system and have salt-retaining properties, whereas glucocorticoids are regulated by adrenocorticotropin (ACTH). Fludrocortisone is the most common mineralocorticoid, and glucocorticoids are further classified into short-acting, intermediate, and long-acting based on the duration of action. Glucocorticoids are commonly used to treat endocrine and non-endocrine disorders, including inflammatory, allergic, immunologic, and malignant conditions [1,2]. Numerous studies have shown that long-term glucocorticoid use, even in low doses, is an independent predictor of various adverse effects. The risk is both dose- and duration-dependent [3].

Dexamethasone is a long-acting, potent glucocorticoid with no mineralocorticoid activity (no salt-retaining properties). It is metabolized in the liver with a half-life of one to five hours with intravenous administration. Neuropsychiatric effects of glucocorticoids, including dexamethasone, comprise depression, mania, agitation, mood lability, anxiety, insomnia, catatonia, depersonalization, delirium, dementia, and psychosis.

In this case report, we describe the onset of acute psychosis, in a patient with no prior history of psychiatric illnesses, associated with the administration of intravenous dexamethasone for a week.

## Case presentation

The patient, Ms. A, was a 55-year-old woman of Caribbean descent with a history of type 2 diabetes mellitus and hypertension with no past psychiatric history. Before admission, she had not been on any medications for her diabetes or hypertension. Ms. A initially presented to the hospital with shortness of breath, intermittent productive cough with white sputum, chest tightness, chills, sporadic headaches, anosmia, loss of taste, and malaise for one week in the context of receiving the first dose of Moderna coronavirus disease 2019 (COVID-19) vaccine (Moderna, Inc, Cambridge, MA) nine days ago. She reported a gradual worsening of the symptoms mentioned above and denied any recent travel or sick contact. On arrival to the emergency room, the patient tested positive for COVID-19 and was noted to have hypoxia with oxygen saturation of 87% and tachypnea with a respiratory rate of 24 bpm. On physical examination, the patient was alert and oriented, in distress, with normal heart rate and regular rhythm with the presence of rhonchi. Motor strength and sensations were intact with no focal neurological deficits.

Laboratory evaluation showed positive severe acute respiratory syndrome coronavirus 2 (SARS-CoV-2), and chest X-ray showed ill-defined bibasilar opacities. EKG showed normal sinus rhythm. She was subsequently admitted and treated for acute hypoxic respiratory failure secondary to COVID-19 pneumonia. During the hospital course, she was noted to have elevated potassium of 5.1 mEq/L (improved to 4.2 mEq/L with Kayexalate), elevated lactate dehydrogenase of 563 U/L, ferritin of 212 ng/mL, and C-reactive protein of 27.2 mg/L. Also noted were elevated prothrombin time of 13.5 seconds, normal activated partial thromboplastin time (aPTT), normal d-dimer levels of 193 ng/mL, low to normal calcium levels from 8.2 to 8.6 mg/dL, elevated glucose of 113 mg/dL, and normal sodium, magnesium, phosphorous, and kidney and liver function tests. White blood cell count was 4.2 x x 10^9^/L with a hemoglobin of 12.5 g/dl, and tests were negative for the respiratory syncytial and influenza viruses. On day one, Ms. A was treated with 3-6 L of oxygen supplementation, dexamethasone 6 mg IV for seven days, remdesivir 100 mg for five days, and deep vein thrombosis (DVT) prophylaxis with enoxaparin 40 mg. She was hemodynamically stable and discharged from the hospital after 11 days of admission on 4 L/minute of supplemental oxygen via nasal cannula. She did not have any psychiatric symptoms during this hospital course.

Two weeks after the initial hospital admission and three days after discharge, Ms. A presented to the hospital with complaints of a one-day history of bizarre abnormal behavior. While in the emergency room, the patient showed agitated behavior, pressured speech, and paranoid grandiose religious delusions. She refused to take medications by mouth given by the hospital and only wanted to take medications brought from home and complained that these were taken away from her by the hospital staff. She reported that she only wanted to take a blood thinner and did not want to take any medications by mouth, as per the wish of Jesus Christ. She said that Jesus Christ would cure her COVID-19 and that Jesus was her savior. On physical exam, Ms. A was alert and oriented to time, place, and person, with normal motor strength and normal heart rhythm and breath sounds. EKG showed sinus tachycardia with a ventricular rate of 120 bpm with premature atrial complexes and voltage criteria for left ventricular hypertrophy with repolarization abnormality. Chest X-ray showed hazy bilateral lung infiltrates, cardiomegaly, and mildly increased pulmonary vascularity. While in the emergency room, she removed the monitoring wires, threw oil on hospital staff, and was given intravenous diphenhydramine 25 mg, haloperidol 5 mg, and lorazepam 2 mg.

Ms. A was then admitted to the inpatient psychiatric unit. MRI of the brain was performed, which was unremarkable. Vitamin B12, rapid plasma reagin (RPR), and HIV tests were also unremarkable, and there were no electrolyte abnormalities. Physical exam findings were unremarkable for infectious cause or focal neurological deficits. During this hospital course, Ms. A showed paranoid behavior with delusions, poor insight and judgment, poor impulse control, and hyperverbal and pressured speech; she was very theatrical, noted to be hugging the TV, religiously preoccupied, delusional, and difficult to redirect. There were also episodes of the patient pacing back and forth, being loud, and yelling and screaming in the hallways.

Ms. A stated that God continued to speak to her in Gospels and that she had the blood of Jesus and that she did not have COVID-19. She reported not sleeping at all for the last three days. Ms. A complained that she had been poisoned about 10 years ago by her sister-in-law and admitted to a hospital; however, her husband denied this. There were no diurnal or nocturnal fluctuations in her paranoid behavior, and she was alert and oriented throughout the hospital course. She denied suicidal or homicidal ideation, intention, plan, auditory or visual hallucinations, or any history of self-harming behavior.

The patient used to work as a certified nursing assistant and worked with hospice patients. The last time she had worked was three weeks ago. Ms. A had been married for about 10 years and had four adult children from her previous marriage. Her brother had also been diagnosed with steroid-induced mania and psychosis a few days ago. There was no other family history of mental health diagnosis. There was also no prior psychiatric history or abnormal behavior as per family members and the patient's pastor.

Ms. A was treated with oral haloperidol 5 mg twice daily for psychosis, sodium valproate 500 mg twice daily for mood stabilization, acetylcholine 1 mg orally twice daily to prevent extrapyramidal symptoms, and clonazepam 1 mg twice daily for anxiety and insomnia. She responded well to milieu therapy and medication management. Her hygiene and grooming improved, and the patient reported feeling better, tolerating the meals, and sleeping better. She denied any active psychotic, manic, or depressive symptoms, and was less religiously preoccupied but continued to pray for humankind to get cured of COVID-19.

Ms. A’s insight and judgment improved, and haloperidol was discontinued and switched to aripiprazole since it had a lesser side effect profile. She was then discharged after four days of hospital stay on oral aripiprazole 10 mg once daily and sodium valproate 500 mg twice daily. Three weeks after discharge, she reported feeling better and did not want to continue these medications. She stopped taking both medications and exhibited no mood changes or psychosis during monthly outpatient follow-ups for three months. 

## Discussion

Glucocorticoids are administered via various routes, including parenteral, oral, intraarticular, nasal inhalation, lung inhalation, topical application, and eye drops, and these have anti-inflammatory, metabolic, and immunogenic effects. The duration of action and the dose equivalents of various glucocorticoids are listed below in Table 1 [4].

**Table 1:** Glucocorticoid dose equivalents and duration of action

Activity	Glucocorticoids	Duration of action (hours)	Dose equivalent (mg)
Short-acting	Hydrocortisone	8-12	20
	Cortisone	8-12	25
Intermediate-acting	Prednisone	12-36	5
	Prednisolone	12-36	5
	Methylprednisolone	12-36	4
	Triamcinolone	12-36	4
Long-acting	Dexamethasone	36-72	0.75
	Betamethasone	>48	0.6

In addition, glucocorticoids are associated with multiple adverse effects affecting various organ systems, as listed in Table 2 [5].

**Table 2:** Adverse effects of corticosteroids

Organ systems	Adverse effects
Neuropsychiatric	Mood changes – mania/depression/dysphoria/euphoria, psychosis, insomnia, seizures, vertigo, headache, increased motor activity (akathisia)
Ophthalmologic	Posterior subcapsular cataract, glaucoma, ocular nerve damage, exophthalmos, ophthalmic fungal, or viral infections
Cardiovascular	Hypertension, fluid retention, thromboembolism, arrhythmias, arteriosclerosis, thrombophlebitis
Gastrointestinal	Nausea/vomiting, gastritis, esophagitis, peptic ulcer disease, steatohepatitis pancreatitis
Endocrine/metabolic	Hyperglycemia, weight gain, secondary adrenal insufficiency
Female reproductive	Amenorrhea, post-menopausal bleeding
Musculoskeletal	Osteoporosis, myopathy, muscle wasting, avascular necrosis of femoral head
Dermatologic	Hirsutism, acne, skin atrophy, striae, ecchymoses/purpura
Hematologic/immune	Leukocytosis, increased susceptibility to infections

Long-term therapy with glucocorticoids results in depression or apathy, whereas short-term use induces psychosis, mood lability, delirium, and other excitatory psychiatric disturbances. These include agitation, anxiety, distractibility, fear, hypomania, insomnia, irritability, lethargy, labile mood, pressured speech, restlessness, and tearfulness. Risk factors for the development of neuropsychiatric issues include higher doses, a dose equivalent to ≥80 mg prednisone. Other risk factors include female gender, age >30 years, history of alcohol use disorder, bipolar disorder, depression, or neuropsychiatric disorders [6,7].

The patient described in the case report developed acute psychosis more than three weeks after receiving the COVID-19 vaccine, two weeks after being diagnosed with acute respiratory failure, and one week after completing a week-long course of intravenous dexamethasone. She did not exhibit any psychiatric symptoms during the initial hospital admission and had no prior psychiatric history.

Psychiatric side effects most commonly occur early in the therapeutic course, within five days of initiation of glucocorticoids, averaging 11.5 days. However, these can occur at any time during steroid therapy and can persist even after stopping the treatment. The most common psychiatric disorders were depression (35%), mania (31%), psychosis (14%), and delirium (13%), based on a retrospective study of 14 cases of steroid-induced psychiatric disorders [8].

Pathophysiology of steroid-induced neuropsychiatric effects

The possible pathophysiology includes glucocorticoid-induced hippocampal dysfunction associated with decreased hypothalamic corticotropin-releasing hormone (CRH) and circulating ACTH hormone levels. Dementia associated with steroid use is attributed to hippocampal and frontal-cortex impairment [9].

The incidence of steroid-induced severe neuropsychiatric effects is variable and was noted in about 5.7% of patients in a meta-analysis of 2,555 patients. The occurrence of any psychiatric symptoms was 18.6% in patients receiving >80 mg/day of prednisone (12 mg/day dexamethasone). Of note, the steroid dose was not predictive of the time of onset, severity, type, or duration of symptoms. Symptoms usually resolved with dose reduction or stopping the steroid. The use of atypical antipsychotics or mood stabilizers was found beneficial as well [10].

Recurrent cases of steroid-induced mood disorder have been reported in patients with a previous history of bipolar disorder. Therefore, the use of antidepressants and mood stabilizers can be considered in these patients [11].

Other possible causes of psychosis

The other possible mechanism for psychosis in this patient was due to SARS-CoV-2 infection. This virus causes COVID-19, affects multiple organ systems, and leads to psychiatric and neurocognitive symptoms [12]. These symptoms include depressed mood, anxiety, insomnia, anger and fear in mild disease, depression and post-traumatic stress disorder in moderate illness, and exacerbation of psychiatric disorders in severe disease [13].

The onset of neuropsychiatric symptoms with COVID-19 is also variable. For example, one case report has discussed a patient with no history of psychiatric illness who developed a delayed onset of manic-like symptoms related to SARS-CoV-2 viral infection on day 17 of the disease [14]. On the other hand, 5-8% of COVID-19 patients had newly diagnosed psychiatric illness between 14-90 days, based on a review of 62,354 COVID-19 cases [15]. Our patient presented with psychosis about 23 days after receiving the COVID-19 vaccine and 21 days after the onset of other COVID-19 symptoms.

Autoimmune mechanisms with the involvement of several immune loci and lymphocyte markers are linked to the pathophysiology of COVID-19 psychosis. These include the major histocompatibility complex, CD4, CD8, CD19, and CD20 [16]. The other mechanism of neuroinflammation in COVID-19 is related to the dysregulation of cytokine systems. Proinflammatory cytokines including interleukin (IL)-6, tumor necrosis factor (TNF)-alpha, IL-8, and IL-10 are triggered in response to COVID-19 infection, resulting in the derangement of the blood-brain barrier [17]. Therefore, the role of testing for inflammatory biomarkers and the use of non-steroidal anti-inflammatory medications for COVID-19-related psychiatric illness needs to be evaluated. In addition, further research may be required to assess the role of the detection of antigens, antibodies, and immune complexes in the cerebrospinal fluid (CSF) of patients with neuropsychiatric manifestations of COVID-19.

The patient was also given remdesivir for five days for COVID-19; however, there have been no reports of remdesivir-induced psychosis [18].

The third possible reason for acute change in mental status with paranoid behavior is delirium secondary to hypoxic respiratory failure. The usual instrumental tools to diagnose delirium include the Confusion Assessment Method for the Intensive Care Unit (CAM-ICU) and the Intensive Care Delirium Screening Checklist (ICDSC). Unfortunately, clinicians often do not recognize delirium. In one study, over 40% of patients referred to a psychiatrist to manage depression were eventually noted to have delirium [19]. Nevertheless, delirium was ruled out by history, physical exam, and laboratory evaluation in our patient.

Given that the patient presented with psychiatric symptoms one week after steroid administration, that is the most likely cause of the patient’s acute psychosis. In addition, the patient's brother had recently been diagnosed with steroid-induced mania and psychosis. However, the other two variables - SARS-CoV-2 viral infection and delirium - present a crucial challenge in diagnosing and treating patients with neuropsychiatric symptoms in the context of steroid use.

## Conclusions

Glucocorticoids are associated with multiple adverse effects impacting various organ systems. This case report highlights the importance of paying close attention to neuropsychiatric side effects in patients treated with corticosteroids. In this case report, we discussed a previously normal patient with no prior psychiatric history who developed acute psychosis in the context of steroid administration. Based on our findings, we recommend that clinicians perform a detailed assessment of all patients presenting with psychiatric symptoms, including evaluating for signs and symptoms of delirium and obtaining a thorough history of current and past medication use. Further research is needed to identify the pathophysiology and predisposing factors contributing to neuropsychiatric side effects of corticosteroid administration. The use of antipsychotics in the management of these sequelae needs to be explored as well.
